# Effect of beinaglutide treatment on weight loss in Chinese patients with type 2 diabetes mellitus and overweight/obesity

**DOI:** 10.20945/2359-3997000000388

**Published:** 2021-07-16

**Authors:** Guiying Wang, Peng Wu, Yan Qiu, Xin Dong, Yingbin Wang, Yanjun Chi, Fengjuan Zhang, Yinyu Li, Jimin Zhang, Zhengli Huang, Xifeng Du, Zhiqiang Du

**Affiliations:** 1 The First Affiliated Hospital of Datong University Department of Endocrinology and Metabolism Datong Shanxi China Department of Endocrinology and Metabolism, The First Affiliated Hospital of Datong University, Datong, Shanxi, China; 2 Shanghai Benemae Pharmaceutical Corporation Shanghai China Shanghai Benemae Pharmaceutical Corporation, Shanghai, China

**Keywords:** T2DM, overweight, obesity, GLP-1

## Abstract

**Objective:**

To evaluate the effect of beinaglutide on weight loss and plasma protein patterns of inflammation/obesity relevant cytokines and biomarkers.

**Materials and methods:**

This study involved 36 adult patients with a body mass index (BMI) of ≥ 24 kg/m^2^ and T2DM. Beinaglutide was administered for three months. Changes in body weight, fasting plasma glucose (FPG) level, 2 h postprandial plasma glucose (2h-PG) level, glycosylated hemoglobin (HbA1c) level, BMI and visceral and subcutaneous fat areas were measured at baseline and after three months of treatment. In addition, relevant inflammation/obesity cytokines and biomarkers were measured.

**Results:**

After three months, beinaglutide treatment led to significant changes, including in body weight, BMI, FPG level, HbA1c level, visceral and subcutaneous fat areas. In addition, serpin E1, leptin, C-reaction protein (CRP) and tumor necrosis factor-α (TNF-α) also decreased significantly. The plasma protein concentrations of CRP (Log2 transformed) were found to be positively correlated with the percentage of weight loss (R = 0.514 and p-value = 0.021).

**Conclusion:**

Beinaglutide treatment resulted in weight loss, plasma glucose control and anti-inflammatory effects in patients with T2DM and overweight/obesity.

## INTRODUCTION

The number of people with overweight/obesity has been steadily increasing. Obesity has been recognized as one of the greatest public health concerns worldwide, especially in developed countries ( 1 ). A study conducted in different parts of China revealed that the prevalence of obesity is increasing ( 2 ). Obesity increases the morbidity of several chronic diseases and also increases the mortality of cardiovascular diseases, diabetes, cancers and musculoskeletal disorders ( 3 – 7 ). Among Chinese patients (aged 35-74 years) with type 2 diabetes mellitus (T2DM) in 2010, 28.3% and 10.1% of males and 31.3% and 16.8% of females were considered overweight and obese, respectively. It is reported that approximately 3.32 million T2DM events were attributable to overweight and obesity ( 8 ).

Several GLP-1 receptor agonists (GLP-1RA) have been promoted for the treatment of T2DM and have multiple potential effects on hyperglycaemia, cardiovascular and liver disease ( 9 , 10 ). Additionally, the mechanism by which GLP-1RA treatment leads to weight loss has been reported ( 11 ). Among these GLP-1RAs drugs, beinaglutide is the only prandial GLP-1 ( 7 –36) receptor agonist with a 100% protein sequence identity to human 7-36 GLP-1. A retrospective observational real-world study reported that beinaglutide significantly reduced the bodyweight of the patients with T2DM and obesity while lowering the plasma glucose and glycosylated hemoglobin (HbA1c) levels ( 12 ).

However, inflammation/obesity relevant cytokines and biomarkers from plasma in patients with T2DM treated with beinaglutide are still unknown. Our study aimed to evaluate the effect of beinaglutide on weight loss and plasma protein patterns of inflammation/obesity relevant cytokines and biomarkers.

## MATERIALS AND METHODS

### Study design and patients

This study was a single-centre, prospective, open-label, self-controlled real-world study (RWS) involving 36 patients with overweight/obesity and T2DM. Patients were recruited from the First Affiliated Hospital of Shanxi Datong University. All patients gave their informed consent before receiving beinaglutide treatment. This study was approved by the Ethics Committee of the First Affiliated Hospital of Shanxi Datong University in China and conducted in accordance with the ethical guidelines of the Declaration of Helsinki (1975).

The inclusion criteria were adult patients (≥18 years old) with a body mass index (BMI) of ≥ 24 kg/m^2^ and a diagnosis of T2DM according to the WHO 1999 criteria. The exclusion criteria were patients with clinically significant cardiac, central nervous system, rheumatic or cancer diseases, females who were pregnant or planning to become pregnant, patients enrolled in another clinical trial within the past three months, patients who used a GLP-1 receptor agonist or weight loss drug in the past three months, patients with long-term use of glucocorticoids that led to being overweight/obese, acute diabetic complications, infections or other endocrine diseases, patients with a history of pancreatitis, cancer of the pancreas, type 2 multiple endocrine neoplasia syndrome, or medullary thyroid carcinoma, or patients with inflammatory intestinal diseases or diabetic gastric paretitis.

Beinaglutide injections were administered twice per day before meals, with a starting dose of 0.06 mg per injection each week, climbing to 0.1 mg per injection for three months. Bodyweight (BW), BMI, visceral fat area (VFA), subcutaneous fat area (SFA), waist circumference (WC), HbA1c level, fasting plasma glucose (FPG) level, 2 h postprandial plasma glucose (2h-PG) level, heart rate and blood pressure were measured at baseline and after three months. Besides, plasma was collected for cytokines and biomarkers detection before and after three months of treatment with beinaglutide. The primary end-point was weight loss after three months compared to the baseline. The secondary end-points were changes in FPG, 2h-PG, HbAlc, BMI, VFA and SFA after three months compared to the baseline.

### Cytokines and biomarkers measurement

Plasma was separated within 24 h from the patients at baseline and three months later and then stored at −80 ˚C for future analysis. The Human Obesity Premixed Mag Luminex Performance Assay Kit (FCSTM08-10, R&D Systems) was used to analyse ten inflammation/obesity related plasma cytokines and biomarkers including Adiponectin/Acrp30, C-Reactive Protein/CRP, Complement Factor D/Adipsin, Serpin E1/PAI-1, CCL2/JE/MCP-1, IL-6, IL-10, Leptin/OB, resistin and TNF-α which sensitivities were 6.4 pg/mL, 1.4 pg/mL, 0.16 pg/mL, 1.8 pg/mL, 0.36 pg/mL, 0.13 pg/mL, 7.69 pg/mL, 0.85 pg/mL, 0.20 pg/mL and 0.60 pg/mL, respectively. The panel is a Luminex system that uses a fluorescent bead system and CV (coefficients of variation) of the cytokines/biomarkers are all below 20%. All assay procedures were performed as described by the manufacturer.

### Quantification of visceral and subcutaneous fat area

Abdominal bioelectrical impedance analysis (BIA) (OMRON, HDS-2000 DUALSCAN) was used to estimate VFA and SFA ( 13 ). The VFA was calculated by calculating the total sectional area of the abdomen with the BIA between the umbilicus and the back.

### Statistical analyses

Statistical analysis was conducted and boxplots were generated using R software (version 3.6.1). A normality test of the original data was performed using shapiro.test function using R software. The cytokines and biomarkers data were log2 transformed to check for normality. Baseline and treatment data from the clinical data set were analysed by paired t-tests based on the normality of the original data. Paired one-side Wilcoxon tests with an alternative hypothesis of “greater” were used for non-parametric tests of the non-parametric plasma cytokines and biomarkers data set. The p-value cut-off was set at 0.05. Correlation analysis was performed using the cor.test function in R software using the Pearson method. Weight loss (weight change) percentage was equal to (baseline body weight (kg)-treatment body weight (kg))/baseline body weight (kg).

## RESULTS

### Baseline characteristics and clinical outcomes

**Figure 1:**
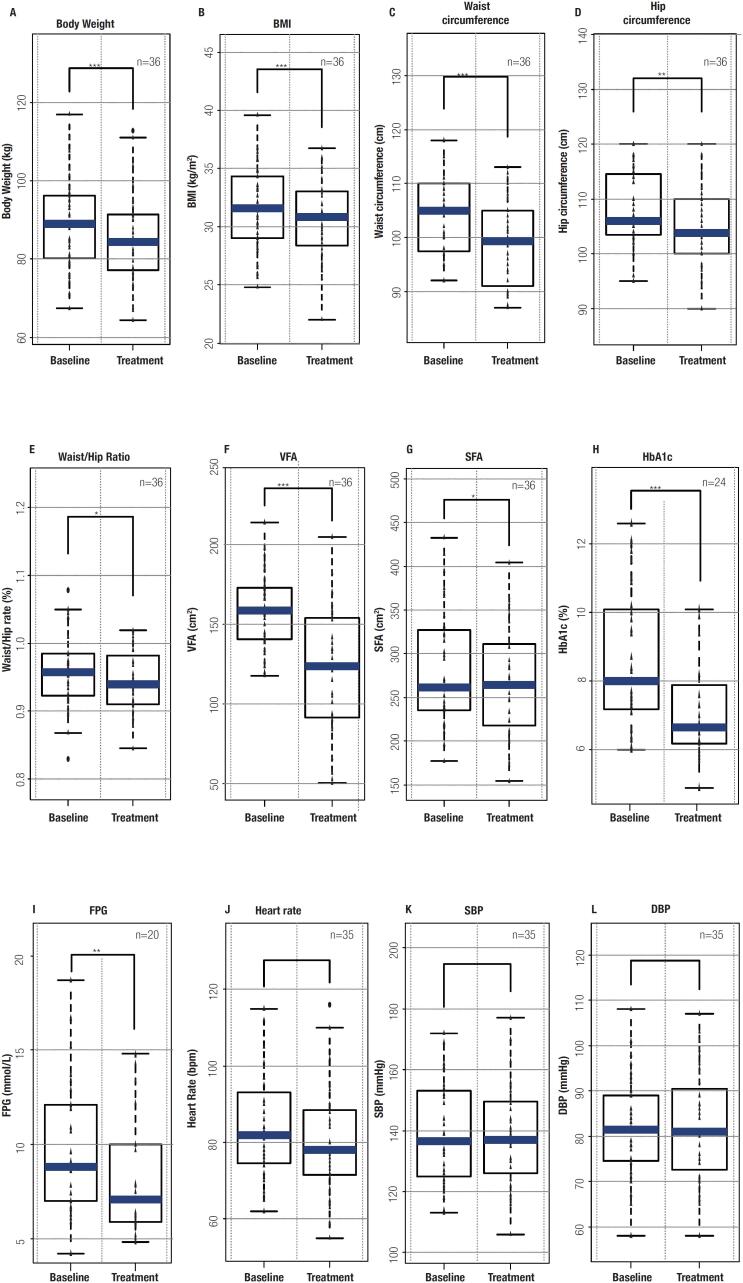
Comparison of baseline and treatment of patients receiving beinaglutide in body weight (A), body mass index (B), waist circumference (C), hip circumference (D), waist/hip rate (E), visceral fat area (F), subcutaneous fat area (G), HbA1c (H), fasting plasma glucose (I), heart rate (J), systolic blood pressure (K) and diastolic blood pressure (L). * p < 0.05; **p < 0.01; ***p < 0.001.

A total of 36 participants complied with the criteria for this study. Twenty-four males and twelve females, with an overall mean age of 46.25 (±13.55) years. Baseline characteristics and clinical outcomes after three months of treatment with beinaglutide are shown in Table 1 and Figure 1 . In brief, bodyweight related physical indices such as BW, BMI, VFA, SFA, WC and hip circumference were reduced significantly after three months (p-value < 0.05). There were 63.9% (23/36) who lost 3% or more in weight, and 41.7% (15/36) lost more than 5% (data not shown). HbA1c and FPG levels were also significantly reduced, while heart rate, systolic blood pressure and diastolic blood pressure did not. These results suggest that patients with T2DM and overweight/obesity could benefit from beinaglutide for weight loss and plasma glucose control.

**Table 1:** Summary of patient characteristics before and after treatment with beinaglutide

		Number	Baseline	Treatment	p-value
Demographics					
	Gender	36	12 F/24 M	12 F/24 M	NA
	Age (y)	36	46.25 (±13.55)	46.25 (±13.55)	NA
Obesity index					
	Weight (kg)	36	88.97 (±11.32)	85.18 (±11.45)	**<0.001**
	BMI (kg/m^2^)	36	31.63 (±3.55)	30.61 (±3.32)	**<0.001**
	HR (bpm)	35	84.06 (±14.01)	79.63 (±13.69)	0.09115
	SBP (mmHg)	35	139 (±16.52)	138.23 (±17.19)	0.68123
	DBP (mmHg)	35	81.42 (±11.21)	81.77 (±12.83)	0.93020
Hip circumference (cm)		36	108 (±7.02)	104.68 (±7.16)	**0.00104**
Waist circumference (cm)		36	103.74 (±7.51)	98.71 (±7.93)	**<0.001**
	VFA (cm^2^)	36	158.81 (±23.11)	120.6 (±38.79)	**<0.001**
	SFA (cm^2^)	36	281.43 (±65.75)	267.64 (±62.88)	**0.02626**
Plasma Glucose test					
	HbA1c (%)	24	8.75 (±2.31)	7.38 (±1.90)	**<0.001**
	FPG (mmol/L)	20	10.01 (±3.96)	8.62 (±3.87)	**0.008**
	2h-PG (mmol/L)	18	14.44 (±4.58)	12.20 (±4.65)	**0.012**

F/M: female/male; BMI: body mass index; HR: heart rate; SBP: systolic blood pressure; DBP: diastolic blood pressure; VFA: visceral fat area; SFA: subcutaneous fat area; HbA1c, haemoglobin A1c; FPG: fasting plasma glucose; 2h-PG: 2-hour plasma glucose. Data are shown as Mean ± SD.

### Inflammation/obesity related cytokines and biomarkers

**Figure 2:**
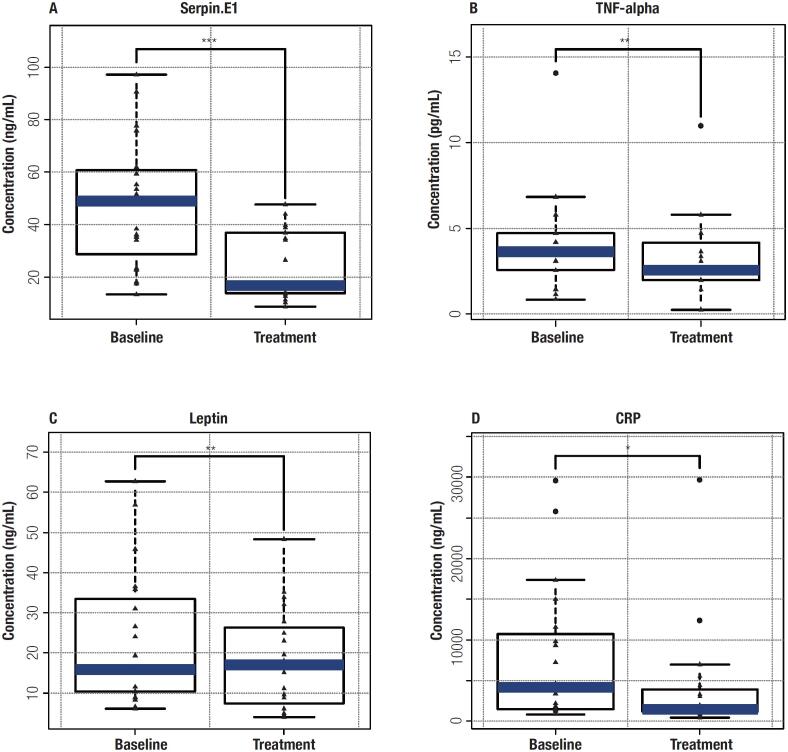
Comparison of baseline and treatment of patients receiving beinaglutide in 4 inflammation/obesity related cytokines/biomarkers. (A) Serpin E1, (B) TNF-α, (C) Leptin, (D) C-reactive protein. * p < 0.05; **p < 0.01; ***p < 0.001.

Plasma concentrations of the panel of ten cytokines and biomarkers were quantified using a Luminex detection platform. Changes compared to baseline and three months of treatment are shown in Table 2 and Figure 2 . Serpin E1, TNF-α, Leptin and CRP were significantly decreased after treatment compared with baseline levels (p-value < 0.05; Figure 2 ). The other five cytokines, adiponectin, CCL2, complement factor D, IL-10 and resistin, were not reduced, while IL-6 failed to be detected due to an extremely low signal. Correlation analysis showed that the Log2 transformed plasma protein concentrations of CRP was positively correlated with the percentage of weight loss (R = 0.514, p-value = 0.021, Table 3 ).

**Table 2:** Changes of inflammation/obesity related plasma cytokines and biomarkers before and after treatment with beinaglutide

	Baseline	Treatment	p-value
Adiponectin (ng/mL)	6046.23 (±4093.7)	6739.87 (±4602.93)	0.938
CCL2 (pg/mL)	121.84 (±49.87)	105.81 (±29.64)	0.084
CRP (ng/mL)	7627.52 (±8437.05)	4197.02 (±6811.5)	**0.044**
Complement Factor D (ng/mL)	6191.26 (±1483.5)	6531.85 (±1774.22)	0.554
IL-10 (pg/mL)	2.07 (±0.25)	2.05 (±0.25)	0.462
Leptin (ng/mL)	23.15 (±16.76)	18.45 (±12.63)	**0.003**
Resistin (pg/mL)	8.48 (±3.52)	9.24 (±4.09)	0.862
Serpin E1 (ng/mL)	48.02 (±24.2)	25.1 (±13.15)	**0.000**
TNF-α (pg/mL)	4.08 (±3.05)	3.26 (±2.55)	**0.005**
IL-6	NA	NA	NA

NA: no available data.

**Table 3:** Correlation analysis between inflammation/obesity related plasma cytokines/biomarkers and weight loss percentage

Cytokines/biomarkers	R	p-value	R-log2	p-value_log2
CRP	0.398	0.082	0.514	**0.021**
Adiponectin	−0.131	0.583	−0.153	0.518
TNF-α	−0.042	0.871	−0.044	0.868
Adipsin	0.109	0.724	0.101	0.742
IL-10	0.061	0.799	0.059	0.806
CCL2	−0.025	0.918	−0.032	0.894
Resistin	**0.546**	**0.013**	0.442	0.051
Serpin E1	0.033	0.89	−0.008	0.973
Leptin	0.176	0.457	0.196	0.407
IL-6	NA	NA	NA	NA

R (and p-value): correlation (and p-value) between weight loss percentage and plasma cytokines/biomarkers concentrations; R-log2 (and p-value_log2): correlation (and p-value) between weight loss percentage and log2 transformed cytokines/biomarkers concentration; NA: no available data.

## DISCUSSION

Beinaglutide was approved as a novel drug for diabetes treatment and the clinical benefit of weight loss and plasma glucose control has also been reported ( 12 ). However, the protein pattern of plasma cytokines and biomarkers are still unclear. This study found a mean weight loss of 3.8 kg and 4.3% mean weight loss percentage after three months of treatment. Moreover, BMI, WC, hip circumference, visceral fat, and subcutaneous fat were also significantly decreased after beinaglutide treatment but not heart rate, diastolic blood pressure or systolic blood pressure (SBP). In addition, HbA1c, FPG and 2h-PG levels were also decreased. It has been reported that heart rate and SBP are affected by other GLP-1RA drugs with a long half-life and continuously activation of GLP-1R ( 14 , 15 ). Therefore, the results of this study indicate the advantages of beinaglutide as a GLP-1 homolog with 100% protein sequence identity to human natural GLP-1 and short half-life (30 min) ( 16 , 17 ).

Abdominal visceral fat and subcutaneous fat are independent risk factors for cardiovascular diseases and metabolic syndrome ( 18 , 19 ). In this study, visceral fat decreased significantly by approximately 24% on average, while subcutaneous fat decreased by about 4% on average after three months of beinaglutide treatment. This finding suggests that beinaglutide primarily reduced body weight through visceral fat, which could be critical for patients with T2DM to reduce the risk of the related diseases.

In addition, we tested the exploratory cytokines and biomarkers of inflammatory or obesity to determine the related factors associated with weight loss after beinaglutide treatment. Serpin E1 is highly expressed in the plasma of patients with obesity and diabetes, indicating a link between them ( 20 ). Our data showed that serpin E1 was significantly downregulated in the plasma of the patients with T2DM and overweight/obesity after three months of treatment with beinaglutide. This finding suggests that the downstream signalling pathway of the GLP-1 receptor might be associated with the regulation of Serpin E1 ( 21 , 22 ).

Obesity is considered a chronic inflammatory disease. Macrophage migration in patients with obesity infiltrates into the vicinity of fat cells, leading to the secretion of inflammatory cytokines, thereby upregulating the expression of TNF-α and resistin ( 23 ). In this study, TNF-α was significantly reduced, suggesting the anti-inflammatory effect of beinaglutide. A parallel study has reported that inflammatory factors such as TNF-α decreased after GLP-1 analog treatment in patients with diabetes and obesity ( 24 , 25 ).

It has been reported that leptin decreased after treatment with diet control or GLP-1 drugs in patients with T2DM with effective or ineffective weight loss ( 25 , 26 ). The serum leptin level in patients with obesity was higher than that in the normal weight group, and a decrease in body weight led to a decrease in leptin levels ( 27 , 28 ). The results in our study are consistent with these studies, thus illustrating the importance of leptin in weight loss.

CRP is a widely investigated biomarker of inflammation in the pathogenesis of several chronic diseases such as cardiovascular disease and diabetes. It has been reported that a decrease in CRP is associated with direct weight loss ( 29 ). Mazidi's study showed that the level of CRP significantly decreased after treatment with GLP-1 RAs ( 30 ). The results of our study verified this finding.

Our research has several limitations. The limited sample size was an inevitable problem in our study, and the study would be informative if there were more data. However, these findings provide a foundation for future clinical research. Next, we will conduct a further study with a large population to validate our findings.

In summary, this study is the first to report the plasma protein pattern of inflammation/obesity related cytokines and biomarkers in Chinese patients with T2DM and overweight/obesity who were treated with beinaglutide. Beinaglutide also decreased bodyweight, as well as the plasma glucose and inflammatory levels. Furthermore, baseline level of CRP is related to the weight loss percentage of beinaglutide treatment in patients with T2DM and overweight/obesity.
